# Inflammatory licensed equine MSCs are chondroprotective and exhibit enhanced immunomodulation in an inflammatory environment

**DOI:** 10.1186/s13287-018-0840-2

**Published:** 2018-04-03

**Authors:** Jennifer M. Cassano, Lauren V. Schnabel, Margaret B. Goodale, Lisa A. Fortier

**Affiliations:** 1 Department of Clinical Sciences, College of Veterinary Medicine, Cornell University, Ithaca, NY 14853 USA; 2 Department of Clinical Sciences, College of Veterinary Medicine, North Carolina State University, 1060 William Moore Drive, Raleigh, NC 27607 USA

**Keywords:** Mesenchymal stem cells, Immunomodulation, Inflammatory licensing, Licensing, Polarization, Regenerative medicine

## Abstract

**Background:**

Inflammatory licensed mesenchymal stem cells (MSCs) have the ability to promote functional tissue repair. This study specifically sought to understand how the recipient tissue environment reciprocally affects MSC function. Inflammatory polarized macrophages, modeling an injured tissue environment, were exposed to licensed MSCs, and the resultant effects of MSC immunomodulation and functionality of the MSC secretome on chondrocyte homeostasis were studied.

**Methods:**

Inflammatory licensed MSCs were generated through priming with either IFN-γ or polyinosinic:polycytidylic acid (poly I:C). Macrophages were polarized to an inflammatory phenotype using IFN-γ. Licensed MSCs were co-cultured with inflammatory macrophages and immunomodulation of MSCs was assessed in a T-cell proliferation assay. MSC gene expression was analyzed for changes in immunogenicity (*MHC-I, MHC-II*), immunomodulation (*IDO, PTGS2, NOS2, TGF-β1*), cytokine (*IL-6, IL-8*), and chemokine (*CCL2, CXCL10*) expression. Macrophages were assessed for changes in cytokine (*IL-6, IL-10, TNF-α, IFN-γ*) and chemokine (*CCL2, CXCL10*) expression. Conditioned medium representing the secretome from IFN-γ or poly I:C-primed MSCs was applied to IL-1β-stimulated chondrocytes, which were analyzed for catabolic (*IL-6, TNF-α, CCL2, CXCL10, MMP-13, PTGS2*) and matrix synthesis (ACAN, *COL2A1*) genes.

**Results:**

IFN-γ-primed MSCs had a superior ability to suppress T-cell proliferation compared to naïve MSCs, and this ability was maintained following exposure to proinflammatory macrophages. In naïve and licensed MSCs exposed to inflammatory macrophages, *MHC-I* and *MHC-II* gene expression was upregulated. The secretome from licensed MSCs was chondroprotective and downregulated inflammatory gene expression in IL-1β-stimulated chondrocytes.

**Conclusions:**

In-vitro inflammatory licensing agents enhanced the immunomodulatory ability of MSCs exposed to inflammatory macrophages, and the resultant secretome was biologically active, protecting chondrocytes from catabolic stimulation. Use of licensing agents produced a more consistent immunomodulatory MSC population compared to exposure to inflammatory macrophages. The clinical implications of this study are that in-vitro licensing prior to therapeutic application could result in a more predictable immunomodulatory and reparative response to MSC therapy compared to in-vivo inflammatory licensing by the recipient environment.

**Electronic supplementary material:**

The online version of this article (10.1186/s13287-018-0840-2) contains supplementary material, which is available to authorized users.

## Background

Mesenchymal stem cells (MSCs) have the therapeutic potential to treat a wide variety of inflammatory and degenerative disease processes in humans and animals [1–5] through their ability to modulate the local tissue environment and stimulate a regenerative healing response [6, 7]. Clinical trials using MSCs have been and are being conducted for the treatment of numerous conditions: diabetes, immune and inflammatory diseases, organ transplantation, and disorders of the lung, liver, kidney, and musculoskeletal system [8]. The horse is a valuable model system for studying and translating MSC therapies to human patients, in particular for application to the musculoskeletal system, because horses naturally develop osteoarthritis (OA) and other orthopedic conditions similar to the human patient. Furthermore, MSCs have been used in the equine clinical setting to treat these orthopedic injuries [9, 10].

Heterogeneity in a stem cell population has been associated with variable therapeutic responses. Inflammatory licensing [11, 12] of MSCs through priming with either IFN-γ or the TLR3 ligand polyinosinic:polycytidylic acid (poly I:C) has been suggested as a means to generate a homogeneous population of immunomodulatory MSCs compared to naïve MSCs, thereby improving the consistency of MSCs as a therapy and in the patient’s response to treatment [13–16]. Priming MSCs with IFN-γ enhances their immunomodulatory function in vitro [13] and markedly improves their ability to treat graft versus host disease [15]. Similarly, priming MSCs with poly I:C improves their immunomodulation ability [17] and their capacity to attenuate symptoms of diabetic neuropathy [16]. MSCs have also been found to acquire immunostimulatory properties through a positive feedback cross-talk loop with natural killer cells [18]. These previous studies focused on how MSCs affect the recipient environment, but little is known about the reciprocal effects—how the recipient environment affects the function of inflammatory licensed MSCs, and whether the MSCs maintain their immunomodulatory capacity when transplanted into an inflammatory recipient environment.

In the present study, we aimed to investigate how the environment of an injured tissue might affect MSC immunomodulatory function. To model an injured recipient tissue environment into which MSCs might be transplanted for regenerative therapy, we used an in-vitro polarized macrophage system to represent the cellular population that is present in tissue sites targeted for MSC therapy [19, 20]. Paracrine signals from the tissue environment regulate the macrophage polarization status, resulting in secretion of numerous cytokines that could positively or negatively alter the function of transplanted MSCs [21]. To gain insight into how inflammatory licensed IFN-γ or poly I:C-primed MSCs might react in an inflammatory recipient environment, and how the primed MSCs affect inflammatory macrophages, primed MSCs and inflammatory macrophages were co-cultured and gene expression changes were studied. As tests of functional significance of the MSC secretome, the immunomodulatory potential of inflammatory licensed MSCs was measured, and their secretome was tested on OA chondrocytes for regulation of genes associated with joint health.

## Methods

### Horses

Fifteen adult horses were used for collection of blood and bone marrow aspirate with approval from the Institutional Animal Care and Use Committee of Cornell University protocol 2006-0026.

### Cell isolation and culture

#### Mesenchymal stem cells

Bone marrow aspirate was collected aseptically from eight horses and the demographics of these horses are presented in Table 1 [22]. Aspirates were cultured using the direct plating method [23] in MSC medium: low-glucose (1 g/dl) DMEM containing 10% fetal bovine serum (FBS, endotoxin concentration < 0.1 EU/ml; Atlanta Biologicals, Flowery Branch, GA, USA), penicillin (100 units/ml), streptomycin (100 μg/ml), and basic fibroblastic growth factor (bFGF, 1 ng/ml). MSCs were expanded and cryopreserved [22] for subsequent experiments. All MSCs were used at passage 4 or lower. A subset of cells was confirmed to undergo trilineage differentiation using the method reported previously by our laboratory [22].

**Table 1:** Demographics of horses for bone marrow aspirate and stem cell isolation

Horse ID	Age (years)	Breed	Sex
111	10	Thoroughbred	Female
112	24	Thoroughbred	Castrated male
113	17	Warmblood	Female
114	14	Paint Cross	Castrated male
115	4	Thoroughbred	Female
116	5	Thoroughbred	Castrated male
117	16	Warmblood	Castrated male
118	1	Thoroughbred	Castrated male

#### Lymphocytes

Lymphocytes were obtained as described previously [22] and stained with 5(6)-carboxyfluorescein diacetate *N*-succinimidyl ester (CFSE; Sigma-Aldrich). They were then suspended in lymphocyte proliferation medium: RPMI 1640 medium (Gibco, Grand Island, NY, USA) containing 10% FBS, 0.1 mM 2-mercaptoethanol, penicillin (100 units/ml), and streptomycin (100 μg/ml). Naive (unstimulated) lymphocytes and lymphocytes stimulated with concavalinA (ConA, 5 μg/ml; Sigma-Aldrich) were used in subsequent immunomodulation (T-cell proliferation) studies.

#### Macrophages

To generate inflammatory macrophages, monocytes were isolated according to previously described protocols [22, 24]. Using MACS selection, CD14-positive (1:100 dilution, mouse anti-equine CD14 antibody, clone 105; courtesy of Dr Bettina Wagner, Cornell University, Ithaca, NY, USA) cells were isolated, counted, resuspended in macrophage media (DMEM containing 10% normal horse serum (NHS; HyClone Laboratories, Logan, UT, USA), l-glutamine (2 mM), penicillin (100 units/ml), and streptomycin (100 μg/ml)), and plated at 1.8 × 10^6^ cells per well in six-well plates. Horse serum was only used in macrophage culture to provide species-specific serum commonly used in macrophage culture experiments [24]. Macrophage media were added every 48 h during culture. Monocytes were allowed to spontaneously differentiate into macrophages over 6–7 days of culture before use in experiments. Using this isolation technique, 99% of cells were confirmed CD14-positive by flow cytometry after 6 days in culture (data not shown). Macrophages were washed with warm phosphate buffered saline (PBS) and then stimulated with IFN-γ (100 ng/ml, recombinant equine interferon gamma; R&D Systems, Minneapolis, MN, USA) for 6 h. Macrophages were washed with PBS, followed by macrophage media, and then were used to stimulate MSCs, or to confirm polarization to an inflammatory macrophage phenotype by gene expression.

### Experimental design

#### MSC priming

MSCs were primed with poly I:C as described previously [17], with modifications in ligand concentration. Based on a series of optimization experiments, 10 μg/ml poly I:C stimulation for 1 h was selected to produce robust changes in gene expression and modulation of mitogen-stimulated T-cell proliferation by MSCs, without affecting MSC viability. MSCs were also primed with 100 ng/ml IFN-γ for 24 h as described previously [13, 15].

#### Co-culture

**Fig. 1:**
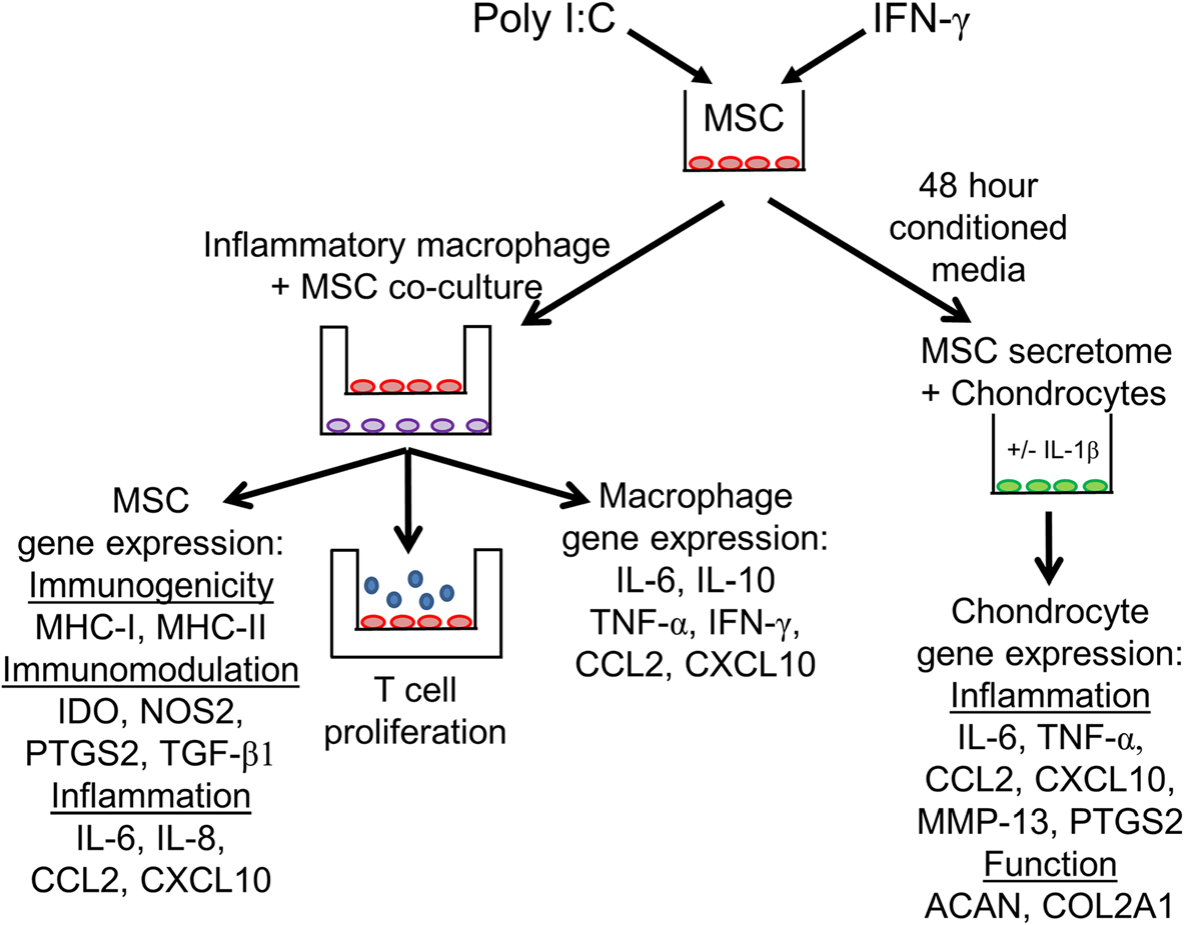
Study design overview. MSCs were primed with polyinosinic:polycytidylic acid (poly I:C) to stimulate TLR3 receptors or interferon gamma (IFN-γ) to induce inflammation. MSCs on co-culture inserts were exposed to proinflammatory macrophages in the bottom of transwells. After co-culture, MSCs were removed, placed in a new well, and lymphocytes were added to allow direct MSC–lymphocyte contact. Gene expression of immunogenicity (*MHC-I, MHC-II*), immunomodulation (*IDO, PTGS2, TGF-β1*), and inflammatory (*IL-6, IL-8, CCL2, CXCL10*) mediators was analyzed. T-cell proliferation assays with mitogen concavalinA (ConA) characterized functional changes in MSC immunomodulation. Macrophage gene expression of cytokines (*IL-6, IL-10, TNF-α, IFN-γ*) and chemokines (*CCL2, CXCL10*) assessed to characterize effect of inflammatory primed MSCs on macrophages. To analyze the functional significance of MSC secretome after priming, MSC conditioned media was generated over a period of 48 h. This primed MSC secretome was then placed on IL-1β-stimulated or untreated chondrocytes, and effect of conditioned media was assessed by measuring inflammatory (*IL-6, TNF-α, CCL2, CXCL10, MMP-13, PTGS2*) and matrix (*ACAN, COL2A1*) gene expression changes in chondrocytes. MSC mesenchymal stem cell, MHC major histocompatibility complex, *IDO* indoleamine 2,3-dioxygenase, *NOS2* inducible nitric oxide synthase, *PTGS2* prostaglandin-endoperoxide synthase 2, *TGF-β1 *transforming growth factor beta-1,* IL *interleukin, *CCL2* C-C motif chemokine 2, *CXCL10* C-X-C motif chemokine 10, *TNF-α* tumor necrosis factor-alpha, *ACAN* aggrecan, *COL2A1* collagen type 2

In the following experiments, MSCs and macrophages used in co-culture were derived from different horses. To assess the effect of priming and/or exposure to inflammatory macrophages on MSC gene expression and immunomodulation function, MSCs were plated on transwell inserts (Corning^®^ Transwell^®^ polyester 0.4 μM pore membrane, six-well plates) and primed with IFN-γ for 24 h, or with poly I:C for 1 h (Fig. 1). MSCs were then washed twice with MSC media, and the inserts were transferred to wells containing inflammatory macrophages. Co-cultures were continued for 6 h, and MSCs were collected for RNA isolation and gene expression analysis as described in “Gene expression analyses,” or transferred to empty wells for use in T-cell proliferation assays. To determine how primed MSCs affect gene expression in inflammatory macrophages, RNA was isolated from macrophages at the end of the co-culture period and analyzed as described in “T-cell proliferation/immunomodulation.” The 6 h co-culture duration was selected after optimization experiments comparing 3 and 6 h of co-culture, based on the production of robust changes in MSC gene expression and modulation of mitogen-stimulated T-cell proliferation by MSCs.

#### Functional significance of primed MSC secretome

To test whether the primed MSC secretome had functional significance in a clinically relevant setting, we used the well-established system of IL-1β stimulation of chondrocytes to generate an OA phenotype. Chondrocytes were isolated from a young adult horse [25] as described previously [26] and plated in chondrocyte media [26], with or without 10 ng/ml IL-1β (recombinant equine IL-1β; R&D) at 200,000 cells/well in a 24-well plate (Fig. 1). After 24 h, the medium was removed and conditioned medium from primed MSCs was added. Stimulation with IL-1β was continued with medium exchange in the IL-1β-stimulated group. After 24 h, chondrocytes were lysed for subsequent RNA analysis.

### Gene expression analyses in MSCs, macrophages, and chondrocytes

Relative gene expression following IFN-γ stimulation was analyzed to confirm polarization of macrophages to an inflammatory phenotype, and macrophage gene expression was also assessed following co-culture with MSCs. *IL-6, IL-8, CCL2*, and *CXCL10* were selected for MSC analysis based on microarray analysis of differentially expressed genes in primed MSCs [27], and on our optimization experiments. Genes associated with inflammatory macrophages include *TNF-α, CCL2, CXCL10*, and *IL-6*, while *IL-10 *is associated with alternatively activated macrophages [21, 28]. *NOS2* is associated with inflammatory macrophages in some species [29], and inflammatory macrophages have also been documented to secrete IFN-γ [30]. Additionally, expression of *MHC-I, MHC-II, IDO, PTGS2 *(also known as cyclooxygenase-2 (COX-2)), and *NOS2* was also assessed to further evaluate the antigenicity and immunomodulatory properties of MSCs. To determine the effect of primed MSC conditioned media on IL-1β-stimulated and untreated chondrocytes, *IL-6, IL-8, CCL2, CXCL10, MMP13, ACAN*, and *COL2A1* were selected for analysis [31].

RNA was isolated from MSCs and chondrocytes according to the manufacturer’s directions (E.Z.N.A.^®^ MicroElute Total RNA Kit; Omega Bio-Tek, Norcross, GA, USA) with an on-column DNase step. RNA was extracted from macrophages according to the manufacturer’s directions (RNeasy Mini Kit; Qiagen, Valencia, CA, USA) with an on-column DNase step. Samples were processed for cDNA synthesis using the iScript™ cDNA Synthesis Kit (Bio-Rad Laboratories, Inc., Hercules, CA, USA) with 250 ng of RNA as template. All cDNA samples had a single band of product from a beta-2-microglobulin PCR [32]. RNA extraction kits were selected based on anticipation of RNA yield.

A quantitative real-time PCR assay based on SYBR Green (Applied Biosystems, Foster City, CA, USA) was run on an Applied Biosystems 7900HT Fast Real Time PCR instrument according to the manufacturer’s instructions. All genes were analyzed with a SYBR Green assay except for *MMP13* and *ACAN*, which were analyzed with a Taqman^®^ primer and probe assay, using 18S as a housekeeping gene and the Taqman^®^ Gene Expression Master Mix (Applied Biosystems) according to the manufacturer’s instructions.

All primers are listed and have been validated previously for the genes of interest *MHC-I* [33], *MHC-II *(Douglas Antczak, personal communication), *IDO* [34], *PTGS2* [35], *NOS2* [34], *IL-6* [35], *IL-8* [36], *IL-10 *[35], *TNF-α *[35], *IFN-γ* [35], *CCL2* [37], *CXCL10* [37], *COL2A1* [38], *MMP-13 *[39], and *ACAN* [39], as well as the housekeeping gene *SCAMP3* [32] (Tables 2 and 3). *SCAMP3* was selected as a housekeeping gene as described by Brosnahan et al. [32]. For SYBR green reactions, determination of a single PCR product was made by DNA melting curve analysis [40]. The cycle threshold (CT) values for duplicate samples were averaged and data were analyzed with the ΔCT method where fold change is expressed as 2^−ΔΔCT^ [41].

**Table 2:** SYBR green primers used for PCR analyses

Gene	Abbreviation	Function	Forward 5′–3’	Reverse 5′–3’
Secretory carrier membrane protein 3	*SCAMP3*	Housekeeping gene	CTGTGCTGGGAATTGTGATG	ATTCTTGCTGGGCCTTCTG
Major histocompatibility complex class I	*MHC-I*	Self-recognition	ACCGTGAGGTCACCCTGA	CTCCGTGTCCTGGGTCA
Major histocompatibility complex class II	*MHC-II*	Antigen presentation	TCCCTATGCTGGGACTTTTC	CGCCAGGCTTCAGATAGAAC
Indoleamine 2,3-dioxygenase	*IDO*	Mediator of immunomodulation	TCATGACTACGTGGACCCAAAA	CGCCTTCATAGAGCAGACCTTC
Prostaglandin endoperoxide synthase 2	*PTGS2*	Mediator of immunomodulation	CAGCATAAACTGCGCCTTTTC	AGGCGGGTAGATCATTTCCA
Inducible nitric oxide synthase	*NOS2*	Mediator of immunomodulation	CCAACAATGGCAACATCAGGT	TGAGCATTCCAGATCCGGA
Interleukin-6	*IL-6*	Cytokine	TGCTGGCTAAGCTGCATTCA	GGAAATCCTCAAGGCTTCGAA
Interleukin-8	*IL-8*	Cytokine	GCTGGCTGTTGCTCTCTTG	CCGAAGCTCTGCAGTAATTCTT
Interleukin-10	*IL-10*	Cytokine	GCCTTGTCGGAGATGATCCA	TTTTCCCCCAGGGAGTTCAC
Tumor necrosis factor alpha	*TNF-α*	Cytokine	AAAGGACATCATGAGCACTGAAAG	GGGCCCCCTGCCTTCT
Interferon gamma	*IFN-γ*	Cytokine	CCAGCGCAAAGCAATAAGTG	GGCCTCGAAACGGATTCTG
C-C motif chemokine 2	*CCL2*	Chemokine	GGCTCAGCCAGATGCAATTA	GCTTTCTTGTCCAGCTGCTT
C-X-C motif chemokine 10	*CXCL10*	Chemokine	GACTCTGAGTGGAACTCAAGGAAT	GTGGCAATGATCTCAACACG
Collagen type 2	*COL2A1*	Cartilage extracellular matrix	TCAAGTCCCTCAACAACCAGATC	GTCAATCCAGTAGTCTCCGCTCTT

**Table 3:** Taqman^®^ primers and probes used in gene expression analysis

Gene	Abbreviation	Function	Forward 5′–3’	Reverse 5′–3’	Probe
18S ribosomal RNA	*18S*	Ribosome structural unit	GGCGTCCCCCAACTTCTT	AGGGCATCACAGACCTGTTATTG	TGGCGTTCAGCCACCCGAGATT
Collagenase 3	*MMP-13*	Matrix metalloproteinase	TGAAGACCCGAACCCTAAACAT	GAAGACTGGTGATGGCATCAAG	CAAAACACCAGACAAATGCGATCCTTCCTTA
Aggrecan	*ACAN*	Cartilage extracellular matrix	GATGCCACTGCCACAAAACA	GGGTTTCACTGTGAGGATCACA	CCGAGGGTGAAGCTCGAGGCAA

### T-cell proliferation/immunomodulation

Following inflammatory priming or co-culture, the immunomodulatory capacity of MSC was assessed by quantifying their ability to inhibit mitogen-stimulated T-cell proliferation. After primed MSC/inflammatory macrophage co-culture, MSCs on transwell inserts were transferred to  fresh wells. Lymphocyte proliferation medium ± ConA was added to the bottom of each well, and 2.5 × 10^6^ lymphocytes ± ConA were added to the transwell insert to allow for cell–cell contact with MSCs. The resultant ratio of lymphocytes to MSCs (approximately 15:1) was based on previously published equine lymphocyte proliferation experiments [22, 34]. Cultures were maintained for 72 h, and media were not exchanged over the 3 days. After culture, lymphocytes were aspirated from the wells, blocked with 10% normal goat serum (NGS), and stained with primary mouse anti-horse CD3 antibody (1:10 dilution, clone UCF6G-3.3; University of California Davis, Davis, CA, USA) and secondary goat anti-mouse allophycocyanin (APC) antibody (BD Biosciences).

Proliferation of T cells was evaluated using CFSE attenuation measured by flow cytometry. Events were gated on forward scatter (FSC) and side scatter (SSC) to exclude debris. CD3-positive cells (T cells) were identified by APC fluorescence. Unstimulated T cells were used to set the boundary of nonproliferating cells, such that all cells to the left (lower fluorescence intensity on fluorescein isothiocyanate (FITC)) of that boundary were determined to be proliferating. The percentage of events in the proliferating T-cell gate was determined and compared to the percentage of all APC-positive events. Differences in lymphocyte mitogen response can be caused by naturally occurring variation between horses and over time in an individual. To account for this, the effect of MSCs on lymphocyte proliferation was calculated by comparing all samples to mitogen-stimulated lymphocytes alone (positive control) from that experiment, which was set at 100% lymphocyte proliferation.

### Statistical analyses

Macrophage gene expression data (2^−ΔΔCT^) were analyzed using a two-sided paired *t* test.

T-cell proliferation data and MSC gene expression data following priming and macrophage exposure (ΔCT values) were analyzed using repeated-measures analysis of variance (ANOVA), with a fixed effect of treatment group and the horse as a random effect in the model, followed by a Tukey HSD test for multiple comparisons. Macrophage gene expression data following MSC co-culture (ΔCT values) and chondrocyte gene expression data following MSC secretome exposure (ΔCT values) were analyzed using repeated-measures ANOVA with a fixed effect of treatment group and the MSC horse source as a random effect in the model, followed by a Tukey HSD. Confidence intervals (95%) were calculated for each macrophage and chondrocyte gene expression group’s 2^−ΔΔCT^ values to determine whether the group was significantly different from the control. Data were tested for normality using a Shapiro–Wilk test. All analyses were performed using JMP Pro 11 software (SAS Institute, Cary, NC, USA), and significance was set at *p* < 0.05.

## Results

### Generation of inflammatory macrophages

This study sought to utilize inflammatory macrophage populations to model an inflammatory recipient environment, such as might be present after injury or surgery. Stimulation of macrophages with IFN-γ resulted in an inflammatory macrophage population. Expression of *CXCL10 *(average 2^−ΔΔCT^ value 5859, range 30–22,584; *p* = 0.0078), *TNF-α* (average 2^−ΔΔCT^ value 6, range 3.5–12; *p* = 0.0078), and * IFN-γ* (average 2^−ΔΔCT^ value 6.8, range 0.6–22; *p* = 0.0391) was significantly upregulated following IFN-γ treatment (*n* = 4 horses). Expression of *IL-10 *(average 2^−ΔΔCT^ value 0.5, range 0.2–1.1; *p* = 0.0038) was significantly downregulated in IFN-γ-stimulated macrophages and there was no change in *CCL2* (average 2^−ΔΔCT^ value 1.5, range 0.7–4.1; *p* = 0.25) or* IL-6* (average 2^−ΔΔCT^ value 1.0, range 0.3–2.0; *p* = 0.95) expression with IFN-γ stimulation.

### Immunomodulatory properties of MSCs after inflammatory priming and exposure to inflammatory macrophages

**Fig. 2:**
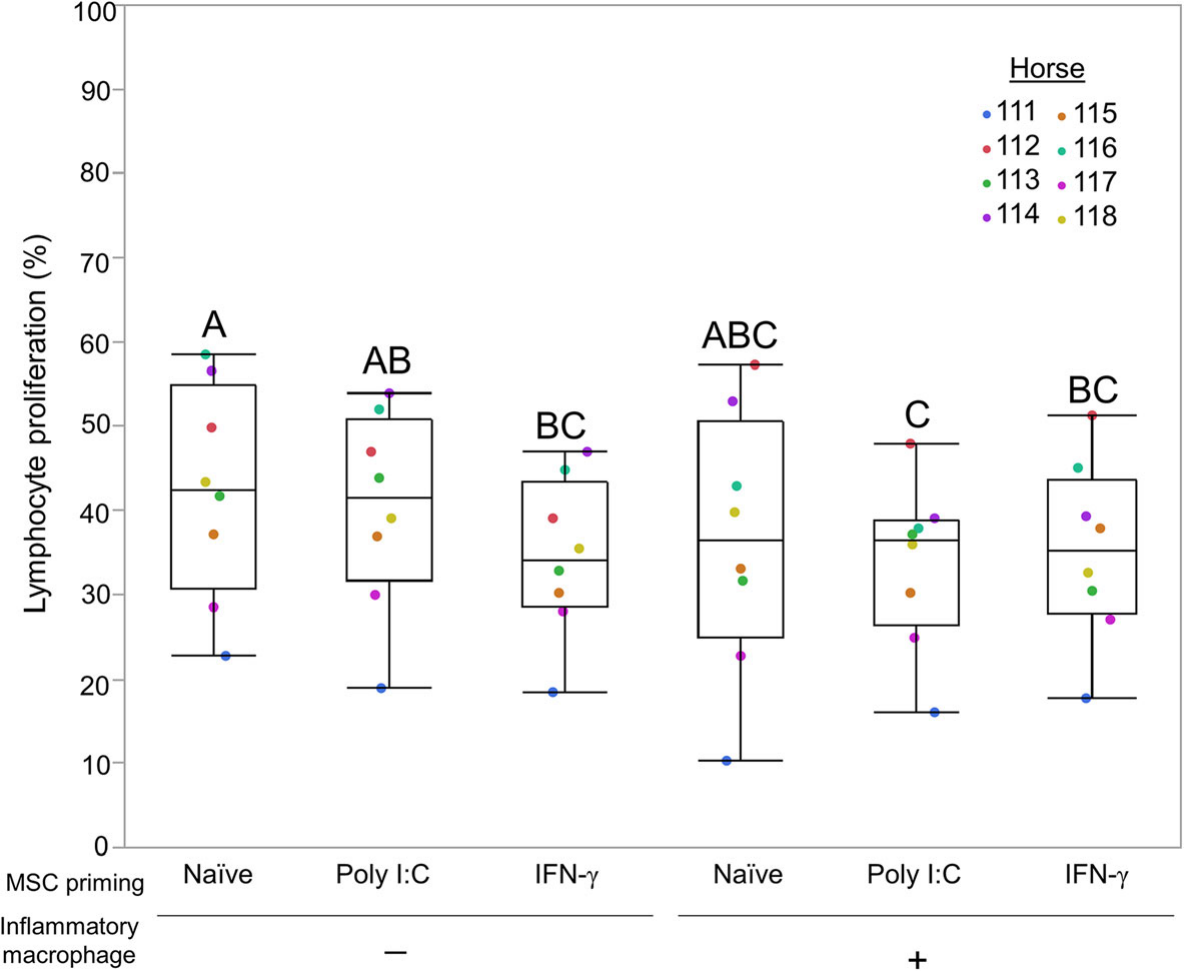
Inflammatory priming and/or exposure to inflammatory macrophages does not impair MSC immunomodulatory function. Ability of MSCs to diminish mitogen-stimulated T-cell proliferation was compared to mitogen-stimulated lymphocytes alone (positive control), which was set to 100% T-cell proliferation. An increase in MSC immunomodulation was found in IFN-γ-primed MSCs, MSCs primed with IFN-γ followed by inflammatory macrophage exposure, and MSC priming with poly I:C followed by inflammatory macrophage exposure. Individual horses denoted by different colored dots. Box and whisker plots represent *n* = 8. Groups not sharing same uppercase letter are significantly different. *p* < 0.05; repeated-measures ANOVA, with horse as a random effect, followed by Tukey HSD test for multiple comparisons. MSC mesenchymal stem cell, poly I:C polyinosinic:polycytidylic acid, IFN-γ interferon gamma

To determine how the immunomodulatory function of inflammatory licensed MSCs changed following exposure to inflammatory macrophages, the ability of MSCs to modulate mitogen-stimulated T-cell proliferation was assessed using a T-cell proliferation assay. Lymphocytes used in these studies were derived from different horses than the MSCs, resulting in an allogeneic study design. The immunomodulatory function of inflammatory primed MSCs, determined by T-cell proliferation assay, was improved or unchanged with exposure to inflammatory macrophages (Fig. 2). MSCs treated with IFN-γ priming alone, or priming MSCs with IFN-γ or poly I:C followed by exposure to inflammatory macrophages, resulted in an increased ability of MSCs to suppress T-cell proliferation compared to untreated MSCs. The immunomodulatory ability of MSCs was not significantly changed with exposure to inflammatory macrophages alone or poly I:C priming alone. No treatment, inflammatory stimuli, and/or macrophage exposure decreased the immunomodulatory capacity of MSCs to suppress T-cell proliferation.

### MSC gene expression after inflammatory priming or exposure to inflammatory macrophages

**Fig. 3:**
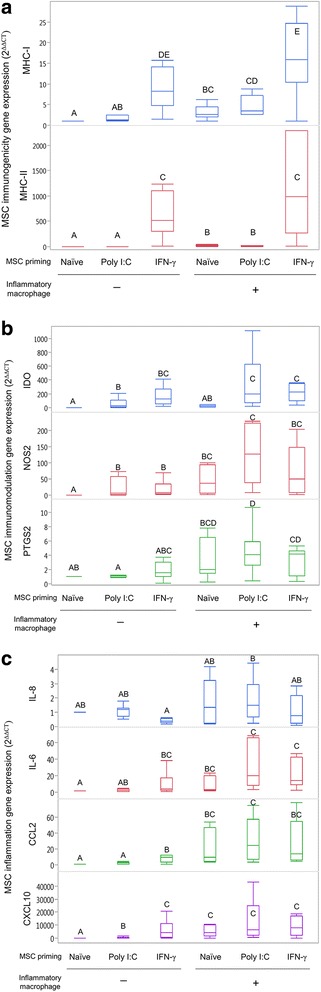
Gene expression changes in MSCs following inflammatory priming and/or inflammatory macrophage exposure. MSCs exposed to polyinosinic:polycytidylic acid (poly I:C) or interferon gamma (IFN-γ), and then to inflammatory macrophages. Resultant changes in expression of (**a**) immunogenicity, (**b**) inflammation, and (**c**) immunomodulatory genes measured and compared to untreated, control MSCs using the 2^–ΔΔCT^ method. Priming with IFN-γ and or inflammatory macrophage exposure induced significant increases in (**a**) *MHC-I, MHC-II*, (**c**) *IL-6*, *CCL2*, and *CXCL10* expression. Priming with poly I:C alone produced few significant changes in gene expression with the exception of (**b**) *IDO*, and sequential exposure to poly I:C then inflammatory macrophages induced significant upregulation of *IDO* compared to inflammatory macrophage exposure alone. Box and whisker plots represent *n* = 8 individual horses. Groups not sharing same uppercase letter are significantly different, *p* < 0.05; repeated-measures ANOVA, with horse as a random effect, followed by a Tukey HSD test for multiple comparisons. MSC mesenchymal stem cell, *MHC* major histocompatibility complex, poly I:C polyinosinic:polycytidylic acid, IFN-γ interferon gamma, *IDO* indoleamine 2,3-dioxygenase, *NOS2* inducible nitric oxide synthase, *PTGS2* prostaglandin-endoperoxide synthase 2, *IL* interleukin, *CCL2* C-C motif chemokine 2, *CXCL10* C-X-C motif chemokine 10

Inflammatory priming with IFN-γ, but not with poly I:C, and exposure to inflammatory macrophages resulted in significant upregulation of MHC class I and II expression in MSCs (Fig. 3a). All primed MSC and macrophage exposure groups, with the exception of inflammatory macrophage exposure alone, resulted in significant induction of *NOS2* and *IDO* expression in MSCs compared to untreated MSCs (Fig. 3b). *PTGS2* expression in MSCs was significantly upregulated in all groups treated with macrophages compared to untreated MSCs (Fig. 3b).

Gene expression of *IL-6*, *CCL2*, and *CXCL10* was significantly upregulated in IFN-γ-primed MSCs and in all MSC groups exposed to macrophages compared to untreated MSCs (Fig. 3c). Following poly I:C priming, *CXCL10* expression, but not *CCL2* and *IL-6* expression, was significantly upregulated in MSCs. Expression of *IL-8* in MSCs was significantly lower following IFN-γ priming compared to poly I:C MSCs exposed to macrophages. There was no significant difference between untreated MSCs and any of the treatment groups in *TGF-β1 *expression (average 2^–ΔΔCT^ value 1.0, range 0.6–1.8).

### Macrophage gene expression in response to inflammatory primed MSCs

**Fig. 4:**
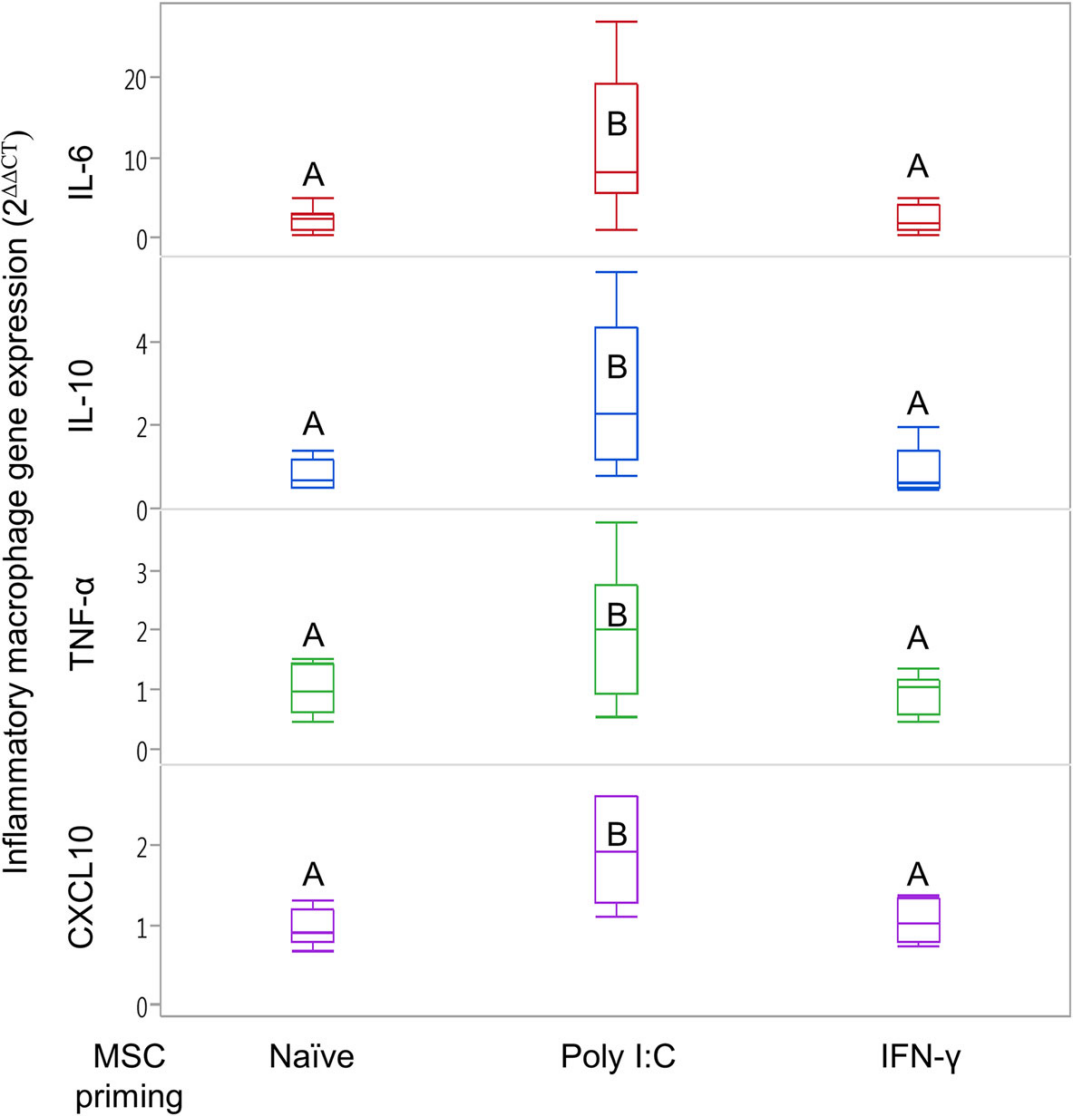
Gene expression changes in inflammatory macrophages following exposure to unstimulated or inflammatory primed MSCs. Inflammatory macrophages co-cultured in a transwell setup with naïve MSCs, MSCs primed with polyinosinic:polycytidylic acid (poly I:C), or MSCs primed with interferon gamma (IFN-γ). Only exposure to poly I:C-stimulated MSCs produced significant changes in macrophage expression of *TNF-α, CXCL10, IL-6*, and *IL-10*. Resultant changes in gene expression measured and compared to control inflammatory macrophages not exposed to MSCs using the 2^–ΔΔCT^ method. Box and whisker plots represent *n* = 8. Groups not sharing same uppercase letter are significantly different, *p* < 0.05; repeated-measures ANOVA, with horse as a random effect, followed by a Tukey HSD test for multiple comparisons. MSC mesenchymal stem cell, poly I:C polyinosinic:polycytidylic acid, IFN-γ interferon gamma, *IL* interleukin, *TNF-α* tumor necrosis factor alpha, *CXCL10* C-X-C motif chemokine 10

Following co-culture of inflammatory macrophages with MSCs in a transwell setup, gene expression of* IL-6, IL-10, CXCL10*, and *TNF-α* increased only in those macrophages exposed to poly I:C-primed MSCs (Fig. 4). Expression of *CCL2* and *IFN-γ* in inflammatory macrophages was not significantly affected by exposure to MSCs (Additional file 1: Table S1).

### The secretome from MSCs diminishes the catabolic effects of interleukin-1B in articular chondrocytes

**Fig. 5:**
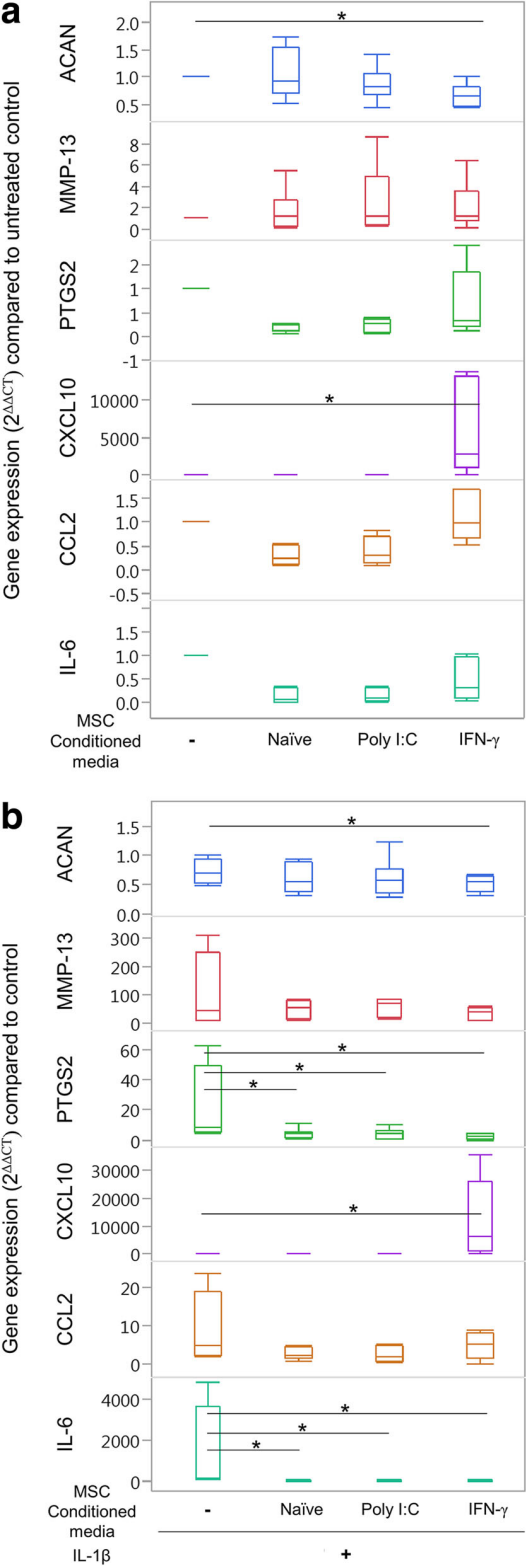
Inflammatory primed MSC secretomes do not induce inflammatory changes in untreated chondrocytes. To analyze functional significance of inflammatory primed MSCs on cartilage health, inflammatory primed MSCs were cultured for 48 h and conditioned media assumed to represent the secretome from primed MSCs. This primed MSC secretome was then placed on (**a**) untreated or (**b**) IL-1 β-stimulated chondrocytes. **a** Conditioned media from IFN-γ-primed MSCs induced downregulation in *ACAN* and upregulation of *CXCL10* compared to untreated control chondrocytes. **b** In IL-1β-stimulated chondrocytes, conditioned media from IFN-γ-primed MSCs also induced downregulation in *ACAN* and upregulation of *CXCL10* compared to untreated control chondrocytes. All conditioned media groups induced downregulation of *IL-6* and* PTGS2 *genes. Box and whisker plots represent *n* = 8 individual horses. *Group significantly different from control IL-1β-stimulated chondrocyte gene expression based on a 95% confidence interval. MSC mesenchymal stem cell, poly I:C polyinosinic:polycytidylic acid, IFN-γ interferon gamma, *ACAN* aggrecan, *MMP-13 *collagenase 3, *PTGS2* prostaglandin-endoperoxide synthase 2, *CXCL10* C-X-C motif chemokine 10, *CCL2* C-C motif chemokine 2, *IL* interleukin

To assess the functional significance of primed MSCs on articular cartilage heath, conditioned medium from primed MSCs was generated to represent the secretome of inflammatory primed MSCs, and used to determine how the inflammatory MSC secretome affects gene expression of arthritic (IL-1β-stimulated) chondrocytes. Conditioned media from inflammatory primed MSCs were used to represent their secretome to test the functional significance of MSCs priming on genes known to be important in chondrocyte homeostasis. Conditioned media from inflammatory primed MSCs induced different changes in chondrocyte gene expression depending on the MSC priming agent and stimulation of chondrocytes with IL-1β (Fig. 5a, b). In non-IL-1β-stimulated chondrocytes, *ACAN* expression was decreased and *CXCL10* expression was increased in response to IFN-γ-primed MSC conditioned media compared to naïve MSC conditioned media (Fig. 5a, Additional file 1: Table S2). *IL-6, CCL2, TNF-α, PTGS2, MMP-13*, and *COL2A1* expression was not significantly different in non-IL-1β-stimulated chondrocytes following exposure to any of the MSC conditioned media treatment groups (Fig. 5a, Additional file 1: Table S2).

Treatment of chondrocytes with IL-1B resulted in 0.3-fold to 0.7-fold decrease in ACAN expression and 7-fold to 300-fold increase in MMP-13 expression compared to untreated chondrocytes, verifying effective catabolic stimulation of chondrocytes. In IL-1β-stimulated chondrocytes (Fig. 5b, Additional file 1: Table S3), ACAN expression was significantly downregulated in IL-1β-stimulated chondrocytes exposed to IFN-γ-primed MSC conditioned media compared to control chondrocytes, and ACAN expression was not significantly different in chondrocytes treated with conditioned media from naïve MSCs, poly I:C-primed MSCs, or IFN-γ-stimulated MSCs. All MSC conditioned media groups reduced expression of PTGS2 and IL-6 in IL-1β-stimulated chondrocytes. CXCL10 expression was significantly increased in response to IFN-γ-primed MSC conditioned media compared to control IL-1β chondrocytes that were not treated with any conditioned medium, and to IL-1β chondrocytes exposed to naïve MSC conditioned media (Fig. 5b). CCL2, TNF-α, MMP-13, and *COL2A1* expression was not significantly different in IL-1β-stimulated chondrocytes following exposure to any of the MSC conditioned media treatment groups, compared to control IL-1β-stimulated chondrocytes (Fig. 5b, Additional file 1: Table S3).

## Discussion

Inflammatory licensing of equine MSCs through priming with IFN-γ resulted in improved immunomodulatory function compared to unstimulated MSCs. This was anticipated and is in agreement with similar studies performed using MSCs from humans and other animal model species. Our results further suggest that when IFN-γ priming of MSCs is followed with exposure to inflammatory macrophages, the enhanced immunomodulation is maintained. This is important because there is opposing evidence of crosstalk between MSC and macrophages in wound repair and cancer studies [42–44], which results in altered phenotype and function. Inflammatory licensing with poly I:C resulted in only modest improvement in MSC immunomodulatory function and gene expression changes compared to that observed with IFN-γ stimulation. This suggests that multiple types of inflammatory signals have the ability to license and improve the immunomodulatory capacity of MSCs [14, 17], but in-vitro inflammatory stimuli priming may produce a more consistent and robust response in MSCs, as opposed to relying on the recipient environment alone to inflammatory license MSCs and elicit a reparative response.

Without priming, the response of MSCs to inflammatory macrophages was highly dependent on the horse from which the MSCs were derived. For example, MSCs from Horse 112 were impaired in their ability to suppress T-cell proliferation following exposure to inflammatory macrophages. However, MSCs from Horses 112 and 118 were exposed to the identical inflammatory macrophage population, and MSCs from Horse 118 responded as anticipated, suppressing T-cell proliferation. This suggests that the unresponsiveness observed in Horse 112 is due to horse–horse MSC variability and not to macrophage differences. The response of Horse 112 could also not be explained by MHC class II expression. Only one horse (Horse 116) had high MHC-II expression at baseline, and this horse’s MSCs responded as expected to macrophage exposure. Interestingly, MSCs from Horse 112 were the upper data points for most of the inflammatory genes measured, further suggesting individual variability in phenotypic response between MSCs from different individuals. The heterogeneity in immunomodulation by MSCs following licensing with IFN-γ and other cytokines is not fully understood and can depend on the experimental methods utilized as well as the inherent variability between donors. Understanding the complex implications of donor and experimental variability in licensing and discovering markers to select immunomodulatory MSCs from a general population will continue to improve the therapeutic potential of MSCs.

Changes in expression of immunostimulatory and immunomodulatory genes have been reported previously in MSCs stimulated with IFN-γ and TNF-α [34]. The results of the present study expand on this concept and demonstrate improved immunomodulatory abilities of MSCs following inflammatory priming with IFN-γ alone, and after sequentially exposing IFN-γ-stimulated MSCs to macrophages. The immunomodulatory ability of IFN-γ-stimulated MSCs exposed to inflammatory macrophages is similar to what has been described previously for MSCs stimulated by both IFN-γ and TNF-α [34]. This is likely the result of TNF-α production by inflammatory macrophages as observed in this study.

Overall, IFN-γ priming appears to induce inflammatory licensing of MSCs to a greater extent than poly I:C priming, and the marked upregulation of inflammatory genes observed in IFN-γ-primed MSCs does not appear to be associated with any deleterious effects of the resultant secretome. The IFN-γ-primed MSC secretome induced similar effects on macrophages and chondrocytes as unstimulated MSCs. Use of primed MSC conditioned media as a surrogate for the secretome did not upregulate catabolic cytokine gene expression, or decrease matrix gene expression in chondrocytes. In IL-1β-stimulated chondrocytes, IFN-γ-primed MSC conditioned media induced a protective reduction of inflammatory gene expression. Previous studies have found the naïve, unprimed MSC secretome to have a protective effect against the inflammatory changes caused by IL-1β in chondrocytes [31], and the results of the present study confirm that inflammatory primed MSCs continued to have a beneficial effect on chondrocytes. The fact that inflammatory licensed MSCs did not have any deleterious effect on chondrocytes, and maintained beneficial anti-inflammatory effects on IL-1β-stimulated chondrocytes, supports the investigation of inflammatory licensed MSCs for clinical use in diseases, such as arthritis. This concept is further supported by previous studies showing that inflammatory synovial fluid alone is insufficient to induce MSC inflammatory licensing [45]. A limitation of this study was the lack of proteomic data to begin to identify which factor(s) from the secretome were responsible for the anti-inflammatory effects on IL-1β-stimulated chondrocytes. As mentioned previously, there are various methods and analyses used in licensing experiments. In an attempt to achieve some standardization in methodology and uniformity in reporting, a 2016 position statement from the International Society for Cellular Therapy advocates a set of analytic approaches including RNA analysis, flow cytometry, secretome analysis, and potency assays to guide future research and inform regulatory agencies [46].

Co-culture of inflammatory macrophages with poly I:C-primed MSCs, but not IFN-γ-primed MSCs, produced the only significant changes in macrophage gene expression. The induction of *IL-10 *and *IL-6* in macrophages following co-culture is a shift from the initial profiles noted after stimulation. Poly I:C-primed MSCs have been found previously to induce a net increase in IL-10 concentration in media from poly I:C-primed MSCs and cancer cells [47]. Poly I:C-stimulated MSCs also upregulated *TNF-α* and *CXCL10* in macrophages, which is a continuation of the trend found after initial IFN-γ macrophage polarization. Since seemingly divergent pathways are induced in the time frame investigated, the regenerative (*IL-10*) versus the proinflammatory (*TNF-α* and *CXCL10*), it is difficult to determine what the net effect of poly I:C-stimulated MSCs is on macrophages.

As expected, priming of MSCs with IFN-γ markedly upregulated expression of *MHC-I* and *MHC-II*, which was further increased by inflammatory macrophage exposure. This increase in MHC expression might translate to an increase in the antigenicity of MSCs, which would be contradictory to allogeneic use in patients. This suggests that regardless of the initial status of MHC-II expression, when placed in an inflammatory environment there is the potential for upregulation of MHC-II expression. This is consistent with observations by our laboratory that when a primary culture of MSCs contains macrophages, the MSCs often have high baseline MHC class II expression. Additionally, our laboratory found that regardless of the initial MHC class II status, donor–recipient equine leukocyte antigen-mismatched MSCs induced an antibody response [48]. This indicates that in-vivo antigenicity of MSCs may be increased in an inflammatory recipient environment, calling into question the time frame that an allogeneic MSC population would have to exert a therapeutic effect prior to targeting by the recipient immune system. For allogeneic purposes, poly I:C inflammatory licensing may be preferred to IFN-γ because it does not appear to increase the antigenicity of MSCs. However, if the in-vitro inflammatory licensing does not induce MSC antigenicity, the inflammatory environment likely will, so perhaps this is not a reason to select one licensing agent over another.

Priming MSCs in vitro could start the process of inflammatory licensing prior to MSCs being transplanted and exposed to a recipient environment. Without in-vitro priming, induction of MSC immunomodulatory function would depend on the environment providing sufficient inflammatory signals. Some reports indicate that MSCs are capable of acting in a proinflammatory manner [17, 47], so relying on the environment to have the necessary signals and in the appropriate time frame in order to activate the immunomodulatory pathway adds a component of uncertainty as to whether or not the transplanted MSCs would be effective. The natural heterogeneity of immunomodulatory function within an individual MSC population has been shown to be reduced with inflammatory priming [13], further supporting the benefits of in-vitro inflammatory priming of MSCs prior to clinical application in regenerative therapies [15, 45].

## Conclusions

In-vitro inflammatory licensing agents enhance the immunomodulatory ability of MSCs exposed to inflammatory macrophages, and the resultant MSC secretome is chondroprotective. Compared to the licensing agents, exposure of MSCs to inflammatory macrophages alone produced an inconsistent change in immunomodulatory function. This suggests that in-vitro inflammatory licensing prior to clinical use could result in more consistent induction of immunomodulatory function, compared to in-vivo inflammatory licensing by the recipient environment alone.

## Additional file

Additional file 1: **Table S1.** Presenting macrophage gene expression following MSC co-culture. **Table S2.** Presenting gene expression in untreated chondrocytes following exposure to MSC conditioned media secretome. **Table S3.** Presenting IL-1β-stimulated chondrocytes gene expression following exposure to MSC conditioned media secretome. (DOCX 16 kb)

## Abbreviations

ACAN: Aggrecan
ANOVA: Analysis of variance
APC: Allophycocyanin
bFGF: Basic fibroblastic growth factor
CCL2: C-C motif chemokine 2
CFSE: Carboxyfluorescein diacetate *N*-succinimidyl ester
*COL2A1*: Collagen type 2
ConA: ConcavalinA
CXCL10: C-X-C motif chemokine 10
FBS: Fetal bovine serum
FITC: Fluorescein isothiocyanate
FSC: Forward scatter
IDO: Indoleamine 2,3-dioxygenase
IFN-γ: Interferon gamma
IL-10: Interleukin-10
IL-1β: Interleukin-l beta
IL-6: Interleukin-6
IL-8: Interleukin-8
MHC: Major histocompatibility complex
MMP-13: Collagenase 3
MSC: Mesenchymal stem cell
NGS: Normal goat serum
NHS: Normal horse serum
NOS2: Inducible nitric oxide synthase
PBS: Phosphate buffered saline
poly I:C: Polyinosinic:polycytidylic acid
PTGS2: Prostaglandin-endoperoxide synthase 2
SCAMP3: Secretory carrier membrane protein 3
SSC: Side scatter
TGF-β1: Transforming growth factor beta-1
TNF-α: Tumor necrosis factor alpha
