# Supplementary material for: Inflammatory licensed equine MSCs are chondroprotective and exhibit enhanced immunomodulation in an inflammatory environment
Source: Stem Cell Res Ther. 2018 Apr 3;9:82. doi: 10.1186/s13287-018-0840-2 (PMC5883371; doi:10.1186/s13287-018-0840-2)
Supplement: Supplementary file 1 — Table S1. Presenting macrophage gene expression following MSC co-culture. Table S2. Presenting gene expression in untreated chondrocytes following exposure to MSC conditioned media secretome. Table S3. Presenting IL-1β-stimulated chondrocytes gene expression following exposure to MSC conditioned media secretome. (DOCX 16 kb) [file 13287_2018_840_MOESM1_ESM.docx]

**Table S1:** Macrophage gene expression following MSC co-culture

| Gene | MSC co-culture group | | |
| --- | --- | --- | --- |
|  | Ctrl MSC | Poly I:C MSC | IFN-γ MSC |
| IL-6 | [1.1,3.5] A | [3.6,18.5]* B | [0.9,3.8] A |
| IL-10 | [0.5,1.1] A | [1.3,4.2]* B | [0.4,1.4] A |
| CCL2 | [1.0,1.5] A | [1.0,1.9] A | [1.0,1.6] A |
| CXCL10 | [0.8,1.1] A | [1.2,3.2]* B | [0.8,1.3] A |
| TNF-α | [0.7,1.3] A | [1.1,2.9]* B | [0.6,1.2] A |
| IFN-γ | [0.2,5.4] A | [0.9,9.7] A | [0.5,4.8] A |

Data are presented as 95% Confidence intervals for 2^ΔΔCT^ values which indicate the fold change from baseline IFN-γ stimulated macrophages. * indicates confidence interval does not contain 1 and thus the change is significant from baseline. Groups that do not share the same letter are significantly different based on delta CT values. P<0.05: repeated measures analysis of variance (ANOVA), with the horse as a random effect in the model, followed by a Tukey HSD test for multiple comparisons.

**Table S2:** Gene expression in untreated chondrocytes following exposure to MSC conditioned media secretome.

| Gene | Conditioned media secretome | | |
| --- | --- | --- | --- |
| NTC Ctrl | Ctrl MSC | Poly I:C MSC | IFN-γ MSC |
| IL-6 | [0,5.4] A | [0,6.9] AB | [0,1.8] A |
| CCL2 | [0,2.0] A | [0,4.5] A | [0.2,3.0] B |
| CXCL10 | [0,3.4] A | [0,415] A | [1021,10871]* B |
| TNF-α | [0.5,1.7] A | [0.2,1.7] A | [0.4,1.8] A |
| COX-2 | [0,3.8] A | [0,1.7] A | [0,1.2] A |
| MMP-13 | [0.1,3.2] A | [0,5.1] A | [0.3,3.8] A |
| Aggrecan | [0.7,1.5] B | [0.6,1.1] AB | [0.5,0.8]* A |
| Col2 | [0.5,2.3] A | [0.3,2.2] A | [0.3,1.5] A |

Data are presented as 95% Confidence intervals for 2^ΔΔCT^ values and indicate the fold change from baseline untreated chondrocytes. * indicates confidence interval does not contain 1 and thus the change is significant from baseline. Groups that do not share the same letter are significantly different based on delta CT values. P<0.05: repeated measures analysis of variance (ANOVA), with the horse as a random effect in the model, followed by a Tukey HSD test for multiple comparisons.

**Table S3:** IL-1β stimulated chondrocytes gene expression following exposure to MSC conditioned media secretome.

| Gene | Conditioned media secretome | | |
| --- | --- | --- | --- |
| IL-1b Ctrl | Ctrl MSC | Poly I:C MSC | IFN-γ MSC |
| IL-6 | [0.1,0.5]* A | [0,0.4]* A | [0.1,0.7]* A |
| CCL2 | [0.1,1.6] A | [0,1.8] A | [0.5,2.8] A |
| CXCL10 | [0.3,2.4] A | [0,286] A | [3566,22353]* B |
| TNF-α | [0,1.2] A | [0,1.3] A | [0,1.6] A |
| COX-2 | [0.3,0.7]* A | [0.1,0.8]* A | [0.2,0.5]* A |
| MMP-13 | [0.6,2.5] AB | [1.0,2.8] B | [0.6,2.0] A |
| Aggrecan | [0.5,1.1] A | [0.5,1.1] A | [0.5,0.9]* A |
| Col2 | [0.5,1.5] A | [0.3,1.8] A | [0,2.1] A |

Data are presented as 95% Confidence intervals for 2^ΔΔCT^ values which indicate the fold change from baseline IL-1β stimulated chondrocytes. * indicates confidence interval does not contain 1 and thus the change is significant from baseline. Groups that do not share the same letter are significantly different based on delta CT values. P<0.05: repeated measures analysis of variance (ANOVA), with the horse as a random effect in the model, followed by a Tukey HSD test for multiple comparisons.
